# Aberrant Long Noncoding RNAs Expression Profiles Affect Cisplatin Resistance in Lung Adenocarcinoma

**DOI:** 10.1155/2017/7498151

**Published:** 2017-11-27

**Authors:** Lijuan Hu, Jian Chen, Fan Zhang, Junjun Wang, Jingye Pan, Jie Chen, Yumin Wang

**Affiliations:** ^1^Department of Laboratory Medicine, The First Affiliated Hospital of Wenzhou Medical University, Wenzhou 325000, China; ^2^Department of Intensive Care Unit, The First Affiliated Hospital of Wenzhou Medical University, Wenzhou 325000, China

## Abstract

**Background:**

Long noncoding RNAs (lncRNAs) have been shown to be involved in the mechanism of cisplatin resistance in lung adenocarcinoma (LAD). However, the roles of lncRNAs in cisplatin resistance in LAD are not well understood.

**Methods:**

We used a high-throughput microarray to compare the lncRNA and mRNA expression profiles in cisplatin resistance cell A549/DDP and cisplatin sensitive cell A549. Several candidate cisplatin resistance-associated lncRNAs were verified by real-time quantitative reverse transcription polymerase chain reaction (PCR) analysis.

**Results:**

We found that 1,543 lncRNAs and 1,713 mRNAs were differentially expressed in A549/DDP cell and A549 cell, hinting that many lncRNAs were irregular from cisplatin resistance in LAD. We also obtain the fact that 12 lncRNAs were aberrantly expressed in A549/DDP cell compared with A549 cell by quantitative PCR. Among these, UCA1 was the aberrantly expressed lncRNA and can significantly reduce the IC50 of cisplatin in A549/DDP cell after knockdown, while it can increase the IC50 of cisplatin after UCA1 was overexpressed in NCI-H1299.

**Conclusions:**

We obtained patterns of irregular lncRNAs and they may play a key role in cisplatin resistance of LAD.

## 1. Introduction

In recent years, a growing proportion of lung adenocarcinoma (LAD) has been diagnosed as non-small cell lung tumor (NSCLC) that is attributable to causes such as environmental pollution. The combination of cisplatin-based chemistry plays an important role in comprehensive treatment program [1]. With the widespread use of cisplatin, tumor cells will inevitably lead to its resistance and the chemotherapy effect was significantly reduced [2]. Studies show that 70–80% of patients can temporarily be alleviated in the initial stage of chemotherapy, but long-term use of cisplatin leads to 60% or more of recurrence rate, and drug resistance rate of recurrent lung tumor was significantly increased and chemotherapy response rate was less than 30% [3]. Nowadays, chemotherapy response rate of patients with advanced LAD was only 30–40% and five-year survival rate less than 15% [4]. According to the survey of American Tumor Society, it was shown that more than 90% of tumor deaths in patients were related to varying degrees of drug resistance. The formation of tumor cells once resistant to cisplatin resulted in multidrug resistance to many first-line chemotherapy drugs, for example, Adriamycin, vinblastine, fluorouracil, and mitomycin, so the harm is particularly serious [5]. Cisplatin resistance is the leading cause of LAD chemotherapy failure, affecting the cure rate and long-term survival rate, seriously affecting the prognosis and quality of life, but also aggravating the social and medical burden. Therefore, it is very important to find the biomarkers and molecular targets related to cisplatin resistance in LAD and then to reverse its resistance to improve the prognosis and to avoid and overcome multidrug resistance.

Recent studies have shown that cisplatin is a nonspecific cell cycle cytotoxic drug; it mainly plays a role of inhibition of tumor cell DNA synthesis [6], inducing apoptosis [7]. The mechanism of cisplatin resistance is very complex and it mainly involved some mechanisms [6, 8–10], including changes in intracellular drug transport (such as ATP binding cassette protein); reducing drug activity interfering with drug action mechanisms such as glutathione (such as GST-pi) can increase cell detoxification function and affect DNA damage repair (breast tumor-associated gene 1, excision repair cross-complementing gene). Genetic changes of the main signal pathways (PDK/Akt, MAPK/Erk, and Wnt) lead to block apoptosis of drug effects. Unfortunately, despite previous advances in genomics and proteomics, the mechanism of cisplatin resistance has not been elucidated.

Studies have shown that lncRNAs known to be aberrantly expressed in normal cells and tumor cells play a role in the regulation of gene expression; so irregular expression of lncRNAs can result in abnormalities of gene expression and tumorigenesis [11–19]. The abnormal expression of lncRNAs is a symbol of many tumors and has been shown to further the development, invasion, and metastasis of tumors by a variety of mechanisms [20, 21]. LncRNAs can regulate expression from the epigenetic, transcriptional, and posttranscriptional levels [20–22].

It was shown that lncRNAs are related to the mechanisms of resistance to cisplatin in tumors, including lung tumor [23–26], providing an important opportunity to elucidate the mechanisms of cisplatin resistance in tumor cells and to find ways to reverse cisplatin resistance. At present, the research of 1ncRNA of cisplatin resistance in LAD is still in its infancy. Some lncRNA molecules, including HOTAIR [27], AK126698 [28], MEG3 [23], H19 [29], and ROR [30], have been screened and identified. It is shown that HOTAIR-mediated LAD cisplatin-resistant mechanism may be through the impact of p21 gene expression to enhance cell apoptosis and G0/G1 phase cell cycle arrest [27], AK126698 regulate non-small cell lung tumor cisplatin resistance partially by Wnt signaling pathway [28], MEG3 expression by inducing mitochondrial apoptosis pathway p53 protein, and Bcl-xl activation of A549/DDP cells to reduce cisplatin resistance [23]. However, lncRNAs of LAD cisplatin resistance need to be further excavated and their mechanisms need to be clarified.

We used a high-throughput microarray to compare the lncRNA and mRNA expression profiles in cisplatin resistance cell A549/DDP and cisplatin-sensitive cell A549. Several candidate cisplatin resistance-associated lncRNAs were verified by real-time quantitative reverse transcription polymerase chain reaction (PCR) analysis. Our results suggest that lncRNA expression patterns may provide new molecular biomarkers for the prediction of cisplatin resistance in LAD.

## 2. Materials and Methods

### 2.1. Cell Lines

A549, NCI-H1299 cells and cisplatin-resistant cell line A549/DDP were cultured in RPMI1640 medium containing 10% fetal bovine serum. The cells were transferred into cell culture flasks by pipetting. The cells were incubated at 37°C in a 5% CO2 incubator, while A549/DDP cell was added to 1 *μ*g/ml of cisplatin maintain the drug resistance. The medium was changed every 2-3 days. The cells were observed under the good condition and the cell will be digested as the cell density was 70%–90%.

### 2.2. RNA Extraction

The test group included three parallel cultured A549/DDP cell and three parallel cultured A549 cell as the control group. Total RNAs of cells were extracted using Trizol reagent (Invitrogen, Carlsbad, CA, USA), based on the manufacturer's protocol. The integrity of the RNA was analyzed by electrophoresis on a denaturing agarose gel. The accurate measurement of RNA concentration (OD_260_), protein contamination (OD_260_/OD_280_ ratio), and organic compound contamination (OD_260_/OD_230_ ratio) was analyzed with a NanoDrop ND-1000 spectrophotometer

### 2.3. Microarray and Computational Analysis

An Agilent Array analysis platform (Agilent Technologies, Santa Clara, CA, USA) was used for microarray analysis,. Slightly, an mRNA-ONLY Eukaryotic mRNA Isolation Kit (Epicentre Biotechnologies, USA) purified mRNA from total RNA after removal of rRNA. Then, each sample was amplified and transcribed into fluorescent cRNA along the whole length of the transcripts without 39 bias with a random priming method. The labeled cRNAs were hybridized onto a Human LncRNA Array v3.0 (8660 K; Arraystar including 30,586 lncRNAs and 26,109 coding transcripts). A specific exon or splice junction probe accurately identified each transcript. For hybridization quality control, the positive probes for housekeeping genes and negative probes were also printed onto the array. The arrays were scanned with an Agilent G2505C scanner after washing the slides, and Agilent Feature Extraction software (version 11.0.1.1) was used to analyze the acquired array images. Quartile normalization and subsequent data analysis were performed with the GeneSpring GX v12.0 software package (Agilent Technologies). Complying with the manufacturer's standard instructions with minor modifications, sample preparation and microarray hybridization were performed [31]. The microarray study was performed by KangChen Bio-tech Corporation, Shanghai, China.

### 2.4. Functional Group Analysis

GO analysis was derived from Gene Ontology (http://www.geneontology.org), which can provide three structured networks of defined terms describing gene product attributes. The *P* value hints the significance of GO Term enrichment in the unmorally expressed mRNA list (*P* ≤ 0.05 was considered obviously significant). We also performed pathway analysis for the unmorally expressed mRNAs based on the latest KEGG (Kyoto Encyclopedia of Genes and Genomes) website. This analysis results allowed us to determine the biological pathways whether a significant enrichment of unmorally expressed mRNAs existed (*P* ≤ 0.05 was considered obviously significant).

### 2.5. Construction of the mRNA-lncRNA Gene Coexpression Network

The coexpression network is constructed and used with calculating a pairwise relation matrix between all probe sets across microarray samples. The result of Pearson relation matrix was transformed into an adjacency matrix [31].

### 2.6. Quantitative PCR

Total RNA was then reverse-transcribed with an RT Reagent Kit (Thermo Scientific, CA, USA), according to the manufacturer's protocols. Total RNA (2 ug) was reverse-transcribed to cDNA. PCR was performed in a total reaction volume of 20 *μ*l, including 10 *μ*l of SYBR Premix (2x), 2 *μ*l of cDNA template, 1 *μ*l of PCR forward primer (10 mM), 1 *μ*l of PCR reverse primer (10 mM), and 6 *μ*l of double-distilled water. The qPCR reaction included an initial denaturation step of 10 min at 95°C; 40 cycles of 5 s at 95°C, 30 s at 60°C, and a final extension step of 5 min at 72°C. The qPCR detected LncRNA with SYBR Premix Ex Taq and an ABI 7200 instrument (ABI Corporation, CA, USA). The candidate lncRNAs were analyzed by qPCR and the sequence information of these gene primers is shown in Table 1. We verified the expression of these lncRNAs by qPCR using GAPDH as a housekeep gene and by calculating 2^−ΔΔCT^ values [32].

### 2.7. Lentivirus-Mediated siRNA, Overexpression Vector Construction, and Transfection

We constructed siRNA GV248 vector targeting UCA1 and overexpression GV303 vector targeting UCA1 (Gene Chem, Shanghai, China). SiRNA sequences were as follows: siRNA1: CCACCTGTAGAGAAGACAAA, siRNA2: GAAGAGTAGAAG ACAGGT, siRNA3: GCCTGGACAAGAACAGT. Transfections were performed by seeding 2 × 10^5^ cells in 6-well plate. After 24 h, the medium was replaced, and the cells were incubated with the transfection complex based on the manufacturer's protocol; the multiplicity of infection (MOI) values was as follows: A549 MOI = 20 and NCI-H1299 MOI = 5. The cells were infected with lentivirus for 72 h, and the siRNA or overexpression efficiency was assessed by qPCR. Puromycin test isolated these cell lines successfully transfected with the lentivirus-mediated vector. The study included NCI-H1299 UCA1 overexpression cell lines (UCA1 OE group), NCI-H1299 was infected with lentivirus negative control LVCON077 vector (NC group), NCI-H1299 (control group), and A549 UCA1 siRNA cell line (UCA1 siRNA group), and A549 was infected with lentivirus negative control LVCON145 vector (NC group) and A549 (control group).

### 2.8. CCK-8 Assays

IC50 (half maximal inhibitory concentration) was detected by Cell Counting Kit-8 (CCK-8, Corning Corporation, UAS) abiding by the manufacturer's protocols. Briefly, 3000 cells were remixed and seeded into a 96-well plate with 10% FBS. The next day, the cells were incubated with CCK-8 for 1 h and the absorbance of 450 nm was analyzed.

### 2.9. Statistical Methods

Statistical analysis was performed for the comparison of two groups in the microarray which was performed with Student's *t*-test and the fold change. The false discovery rate (FDR) was calculated for correcting the *P* value. The threshold value used to designate abnormally expressed lncRNAs and mRNAs according to a fold change of ≥2.0 or ≤0.5 (*P* < 0.05). Differences with *P* < 0.05 were considered statistically significant.

## 3. Results

### 3.1. Overview of lncRNA Profiles

**Figure 1:**
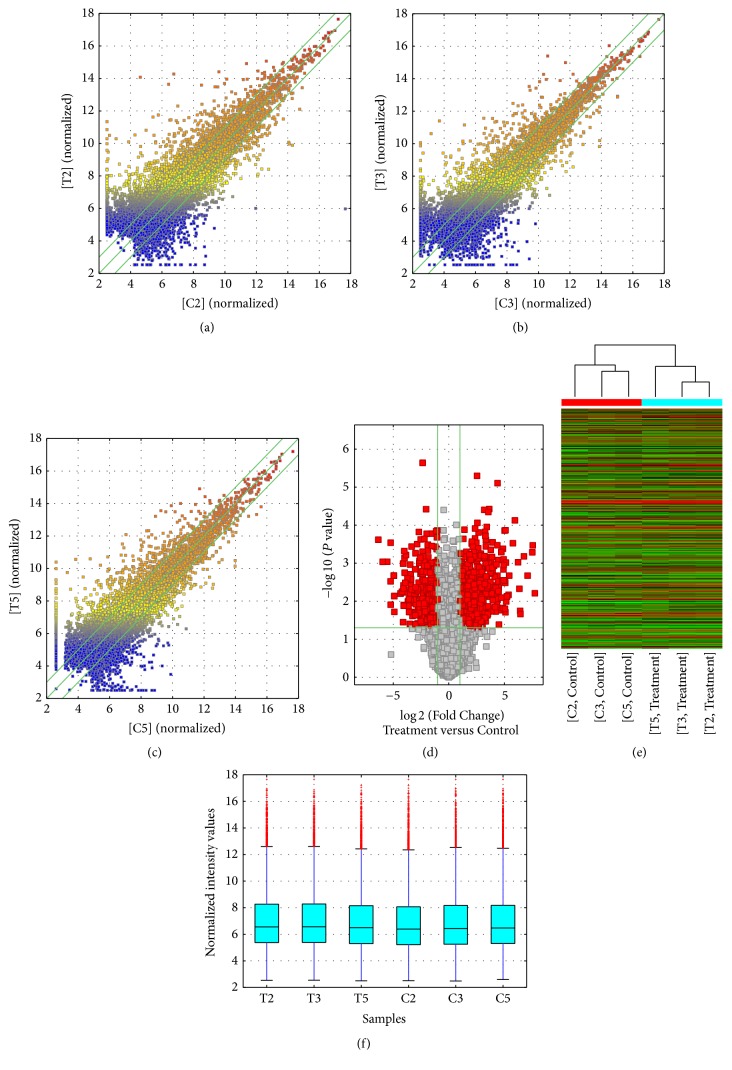
Box plots, scatter plots, and heat map showing the variation in lncRNA expression between the A549/DDP and A549 arrays. The values of the *X* and *Y* axes in the scatter plot are averaged normalized values in each group (log⁡2-scaled). The lncRNAs above the top green line and below the bottom green line are those with a >3-fold change in expression between tissues. (a) Scatter plots of T2 group versus C2 group. (b) Scatter plots of T3 group versus C3 group. (c) Scatter plots of T5 group versus C5 group. (d) Volcanic map, (e) heat map and hierarchical clustering of lncRNA, and (f) box plots showing the distribution of the lncRNA.

The result showed that there were 1,543 differentially expressed lncRNAs between A549/DDP and A549 cell. Among these, compared to A549 group, the 984 lncRNAs upregulated more than twofold in the A549/DDP group, while 559 lncRNAs downregulated (Supplemental Table 1 in Supplementary Material available online at https://doi.org/10.1155/2017/7498151 and Figure 1). These lncRNAs might play an important role in the cisplatin resistance of LAD.

### 3.2. LncRNA Classification and Subgroup Analysis

There were 43 differentially intergenic lncRNAs (LincRNAs) (including 31 upregulated and 12 downregulated) expressed (fold change ≥ 2.0, *P* < 0.05) between A549/DDP cell and A549 cell. We also found some nearby coding genes that may be regulated by these LincRNAs (Supplemental Table 2). LncRNAs with enhancer-like functions (lncRNA-a) were identified with GENCODE annotation. There were 33 lncRNA-a (including 17 upregulated and 16 downregulated) differentially expressed between A549/DDP and A549 cell. We also found some nearby coding genes that may be regulated by these lncRNA-a (Supplemental Table 3). Otherwise, we also found 52 antisense lncRNAs (including 21 upregulated and 31 downregulated) (Supplemental Table 4).

### 3.3. Overview of mRNA Profiles

**Figure 2:**
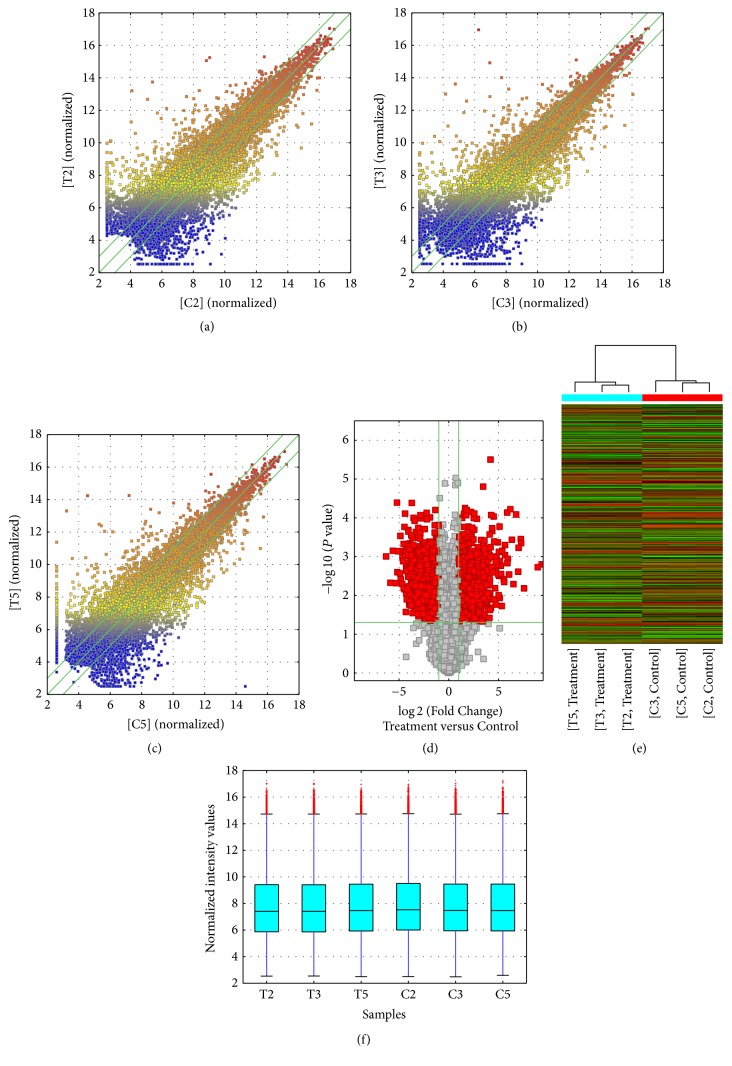
Box plots, scatter plots, and heat map showing the variation in mRNA expression between the A549/DDP and A549 arrays. The values of the *X* and *Y* axes in the scatter plot are averaged normalized values in each group (log⁡2-scaled). The mRNAs above the top green line and below the bottom green line are those with a >3-fold change in expression between tissues. (a) Scatter plots of T2 group versus C2 group, (b) Scatter plots of T3 group versus C3 group. (c) Scatter plots of T5 group versus C5 group. (d) Volcanic map. (e) Heat map and hierarchical clustering of lncRNA. (f) Box plots showing the distribution of the lncRNA.

In total, 1,712 mRNAs were found to be differentially expressed between the A549/DDP and A549 cell, including 795 mRNAs upregulated and 917 mRNAs downregulated (Table 2 and Figure 2). These mRNAs might play a role in the cisplatin resistance of LAD.

### 3.4. GO Analysis

**Figure 3:**
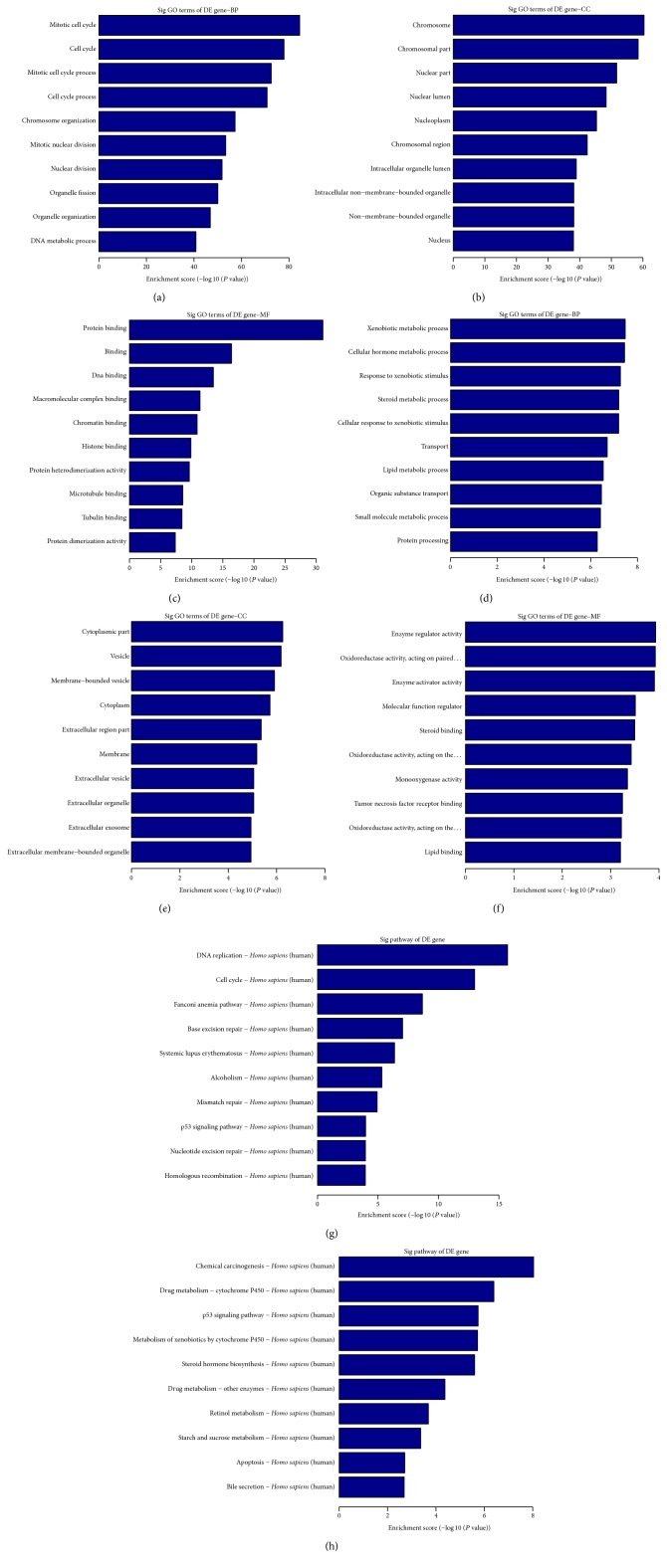
GO and pathway analysis of mRNA from cisplatin resistance in lung adenocarcinoma. (a) Biological processes of downregulated mRNA. (b) Cellular components of downregulated mRNA. (c) Molecular functions of downregulated mRNA. (d) Biological processes of upregulated mRNA. (e) Cellular components of upregulated mRNA. (f) Molecular functions of upregulated mRNA. (g) Pathway analysis of downregulated mRNA from cisplatin resistance in lung adenocarcinoma. (h) Pathway analysis of upregulated mRNA from cisplatin resistance in lung adenocarcinoma.

The genes corresponding to downregulated mRNAs included 979 genes involved in biological processes, 120 genes involved in cellular components, and 137 genes involved in molecular functions (Figures 3(a)–3(c)). The genes corresponding of upregulated mRNAs included 558 genes involved in biological processes, 93 genes involved in cellular components, and 77 genes involved in molecular functions (Figures 3(d)–3(f)).

### 3.5. Pathway Analysis

The 30 upregulated pathways were found, including chemical carcinogenesis, drug metabolism, and p53 signaling pathway (Figure 3(g) and Table 3). 37 downregulated pathways were identified, like DNA replication, cell cycle, Fanconi anemia pathway, and so on. (Figure 3(h) and Table 3). These pathways might play a role in the cisplatin resistance of LAD.

### 3.6. LncRNA-mRNA Coexpression Network

**Figure 4:**
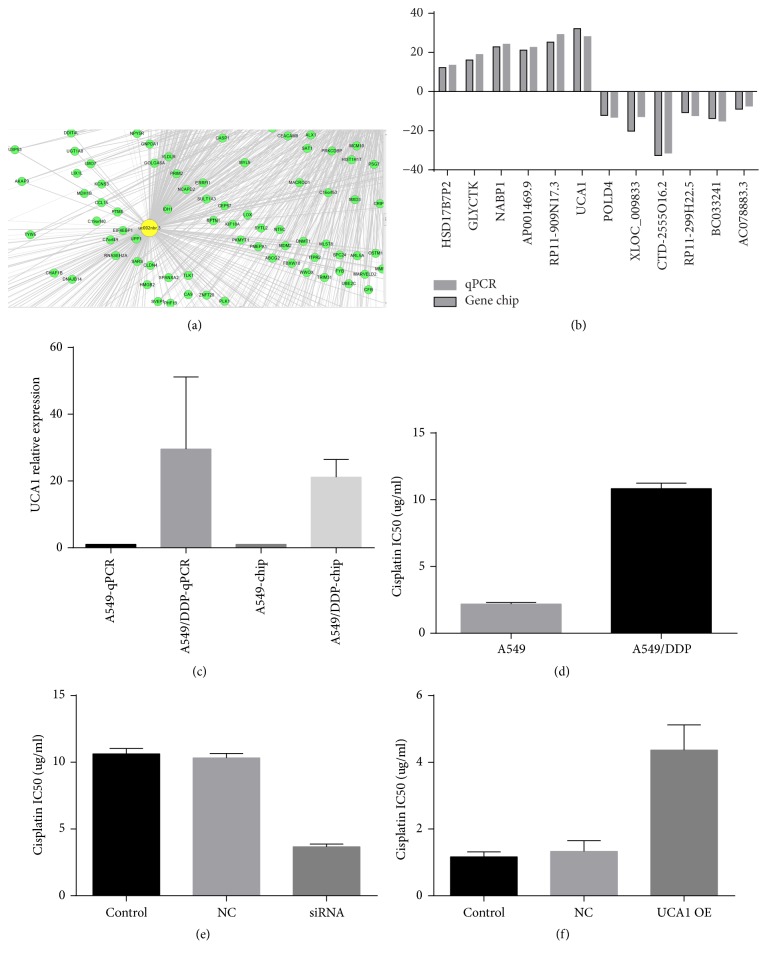
Some lncRNA expression in LAD and A549/DDP, A549 cell. (a) LncRNA-mRNA coexpression network, the results imply that UCA1, ENST00000443252, ENST00000510562, ENST00000565689, ENST00000558690, ENST00000397340, ENST00000440955, and ENST00000507916 are also closely related to many mRNA molecules. (b) Comparison between gene chip data and qPCR result. The validation results of the 12 lncRNAs indicated that the microarray data correlated well with the qPCR results. UCA1 expression level in LAD and A549/DDP, A549 cell. (c) The expression of UCA1 in cisplatin-resistant A549/DDP cells was significantly higher than that in cisplatin-sensitive A549 cells. (d) We used CCK-8 method to detect the sensitivity of A549/DDP cells to cisplatin. (e) The IC50 of cisplatin in UCA1 siRNA group was significantly lower than that in NC group, control group. (f) The IC50 of cisplatin in UCA1 overexpression group was significantly higher than that in NC group, control group.

We build the lncRNA-mRNA coexpression network. See Figure 4(a). The results imply that UCA1 (uc002nbr.3), ENST00000443252, ENST00000510562, ENST00000565689, ENST00000558690, ENST00000397340, ENST00000440955, ENST00000507916 are closely related to many mRNAs and they together prompted resistance of cisplatin in LAD.

### 3.7. Real-Time Quantitative PCR Validation

Based on the features (such as fold difference, gene locus, and nearby encoding gene, and so on.) of the differentially expressed lncRNAs, we initially identified a number of interesting candidate lncRNAs for further analysis (including HSD17B7P2, GLYCTK, NABP1, AP001469.9, RP11-909N17.3, UCA1, POLD4, XLOC_009833, CTD-2555O16.2, RP11-299H22.5, BC033241, and AC078883.3). We found that the microarray results for several of the lncRNAs were consistent with the results of RT-PCR (Figure 4(b)). Of these, UCA1 exhibited significantly changed expression in 20 samples from A549/DDP and A549 cell. The expression of UCA1 in cisplatin-resistant A549/DDP cells was significantly higher than that in cisplatin-sensitive A549 cells (*t* = 71.14, *P* = 0.0002, Figure 4(c)). These results suggest that UCA1 and candidate lncRNAs may play an important role in cisplatin resistance in LAD.

### 3.8. UCA1 Significantly Reduces the IC50 of Cisplatin in A549/DDP Cell after Knockdown

We used CCK-8 method to detect the sensitivity of A549/DDP cells to cisplatin. The results showed that the IC50 of A549 was 2.09 *μ*g/ml ± 0.08 *μ*g/ml, IC50 of A549/DDP was 10.7 *μ*g/ml ± 0.28 *μ*g/ml, and the resistance index was 5.2 (Figure 4(d)). The IC50 of cisplatin in UCA1 siRNA group was significantly lower than that in NC group (*t* = 17.51, *P* < 0.0001), control group (*t* = 37.65, *P* < 0.0001). The IC50 of A549/DDP cells decreased from 10.7 *μ*g/ml ± 0.28 *μ*g/ml to 3.6 *μ*g/ml ± 0.12 *μ*g/ml after UCA1 knockdown, as shown in Figure 4(f). The results showed that UCA1 siRNA, A549/DDP cisplatin resistance can be significantly reversed.

### 3.9. UCA1 Overexpression Significantly Increased the IC50 of Cisplatin in NCI-H1299 Cell

The results showed that the IC50 of cisplatin in UCA1 OE group was significantly higher than that in NC group (*t* = 23.21, *P* < 0.0001), control group (*t* = 29.34, *P* < 0.0001). The IC50 of NCI-H1299 cells increased from 1.20 *μ*g/ml ± 0.04 *μ*g/ml to 4.5 *μ*g/ml ± 0.13 *μ*g/ml after UCA1 was overexpressed, as shown in Figure 4(f). The results showed that UCA1 was overexpressed; NCI-H1299 cisplatin resistance can be significantly increased.

## 4. Discussion

LncRNAs are involved in many biological processes, as X-chromosome inactivation, gene imprinting [33, 34]. Otherwise, lncRNAs are important factors in the control of gene expression in tumor [35] and play an important role in the development, progression, and drug resistance of tumors [36]. Recently, disease-lncRNA association prediction is a recent trend for the identifying potential disease-related lncRNAs [37, 38]. Developing powerful computational models for potential disease-related lncRNAs identification would benefit biomarker identification and drug discovery for human disease diagnosis, treatment, prognosis, and prevention [37, 38].

In this study, we analyzed lncRNA abnormal expression profiles and ascertained the potential role of cisplatin resistance in LAD. High-throughput microarray techniques revealed a variety of differentially expressed lncRNAs, including 984 lncRNAs upregulated and 559 lncRNAs downregulated in A549/DDP cell compared to A549 cell. LncRNAs are usually divided into five categories: sense, antisense, bidirectional, intronic, and intergenic. LncRNAs are known to function by a variety of mechanisms. However, a common and important function of lncRNAs is to change the expression of nearby mRNAs by influencing process of transcription [39] or directly playing an enhancer-like role [40, 41]. In the study, we increased the accuracy of target prediction by comparing abnormally expressed mRNAs with differentially expressed lncRNAs. The expression profiles of 43 intergenic lncRNAs (lincRNAs) hinted that they were differentially expressed between A549/DDP cell and A549 cell. Among these, 31 were upregulated and 12 were downregulated. The expression profiles of 33 lncRNA-a indicated that they were differentially expressed between A549/DDP and A549 cell. Among these, 17 were upregulated and 16 were downregulated. Otherwise, we found 52 antisense lncRNAs, among these, 21 were upregulated and 31 were downregulated. So as to get insights into lncRNA target gene function, GO analysis and KEGG pathway annotation were applied to the lncRNA target gene pool. GO analysis uncovered that the number of genes corresponding to downregulated mRNAs was larger than that relating to upregulated mRNAs. KEGG annotation unveiled that there were 30 upregulated pathways (including chemical carcinogenesis, drug metabolism, and p53 signaling pathway) and 37 downregulated pathways (including DNA replication, cell cycle, and Fanconi anemia pathway). These pathways might be involved in the occurrence and development of cisplatin resistance in LAD.

We found that 12 of the lncRNAs identified in the microarray analysis were confirmed by qPCR to be aberrantly expressed in A549/DDP cell. Among these lncRNAs, UCA1 was the significantly upregulated. Furthermore, we built the lncRNA-mRNA coexpression network and it is shown that UCA1 and some lncRNAs were individually related to some mRNAs; it hinted that lncRNA-mRNA coexpression network might contribute to the development of cisplatin resistance in LAD. UCA1 has also been reported to be related to cisplatin resistance in bladder carcinoma [42, 43]. The expression of UCA1 in A549/DDP cells was significantly higher than that in A549 cells, suggesting that UCA1 may play an important role in cisplatin resistance in LAD. Subsequently, we found the IC50 of A549/DDP cells decreased from 10.7 *μ*g/ml ± 0.28 *μ*g/ml to 3.6 *μ*g/ml ± 0.12 *μ*g/ml after UCA1 knockdown, while the IC50 of NCI-H1299 cells increased from 1.20 *μ*g/ml ± 0.04 *μ*g/ml to 4.5 *μ*g/ml ± 0.13 *μ*g/ml after UCA1 was overexpressed; it hinted that UCA1 might contribute to the development of cisplatin resistance in LAD and further study of the biological function of UCA1 will be required to confirm this notion.

Our study revealed a set of lncRNAs with differential expression from cisplatin resistance in LAD. Furthermore, potential roles for these lncRNAs in the regulation of chemical carcinogenesis and DNA replication signaling pathways were identified. Moreover, we found that UCA1 might contribute to the development of cisplatin resistance in LAD.

## Supplementary Material

Supplemental TABLE 1: Some up-regulated or down-regulated lncRNA in A549/DDP.
Supplemental TABLE 2: Some up-regulated or down-regulated lincRNA in A549/DDP and regulation mRNA.
Supplemental TABLE 3: Some up-regulated or down-regulated enhancer lncRNA in A549/DDP and regulation mRNA.
Supplemental TABLE 4: Some up-regulated or down-regulated antisense lncRNA in A549/DDP and regulation mRNA.

## Figures and Tables

**Table 1:** LncRNAs gene primers in the study for qPCR.

LncRNA gene	Sense primer (5′-3′)	Antisense primer (5′-3′)	PCR product length (bp)
GLYCTK	CGTGCTGATCTCAGGTGGTGA	CTTCACAGAACGTGGCAGGG	164
HSD17B7P2	GTCAGCAACCTGCAGTCATTC	GAGGCTCCAGTTCCCGAATC	283
AP001469.9	TCACACAACCACATCTCGTG	TTGGTCTAAGACTGTTGCCAAG	299
NABP1	GGAGGGTGGGAAGCTTTGAC	CTCCGATCTCATCCCACACG	260
RP11-909N17.3	AGACCCCTGCTATTCCCAGT	AAGGGATGCAGGCAGTTCTC	102
UCA1	ACGCTAACTGGCACCTTGTT	CTCCGGACTGCTTCAAGTGT	124
POLD4	GCACCGTCTCTGGCATCTC	GTTGAGCCTCTGACACCTCC	180
XLOC_009833	AGCCCCTTTATCACTGTGGC	GACATTCAGGAGACGACGGG	147
CTD-2555O16.2	GAGAGAAGGTCCCTTGGTGC	CAGTGCTGCGTTTAGTCATGT	79
RP11-299H22.5	AGTCGCCTTTTCCCTTAGCC	GCAGCTCTCATCTGGTGCTT	108
BC033241	TCTACACAACGCCAGCACAT	TTGACACGTGCTTGGTGAGA	107
AC078883.3	GTGGCAACATCCCTACACCA	ACAGGTTCGTGTTCCCAGTC	245
GAPDH	TGACTTCAACAGCGACACCCA	CACCCTGTTGCTGTAGCCAAA	121

**Table 2:** Some upregulated or downregulated mRNAs in A549/DDP.

Probe name	Fold change	Regulation	Gene symbol
ASHGA5P001180	39.0517312	up	USF2
ASHGA5P016060	3.9089125	up	EFTUD2
ASHGA5P005374	3.2721382	up	TTC39C
ASHGA5P034316	3.2788507	up	ZNF836
ASHGA5P006930	2.1690486	down	TUBD1
ASHGA5P050222	4.1895085	down	PAFAH1B3
ASHGA5P006058	9.1726844	down	TACC3
ASHGA5P010515	2.6021545	down	QKI
ASHGA5P037999	2.193611	down	FAM159A
ASHGA5P009559	2.2639906	down	DIAPH2
ASHGA5P001190	3.9636124	up	CPA4
ASHGA5P002598	2.1978303	up	SNX24

**Table 3:** Pathways analysis of mRNA of A549/DDP cell.

Pathway ID	Definition	Fisher *P* value	Count	FDR	Enrichment_Score
hsa03030	DNA replication, *Homo sapiens *(human)	2.51659*E* − 12	36	7.39876*E* − 10	11.599188
hsa04110	Cell cycle, *Homo sapiens *(human)	6.86608*E* − 11	124	1.00931*E* − 08	10.163291
hsa04115	p53 signaling pathway, *Homo sapiens *(human)	1.51925*E* − 10	68	1.48887*E* − 08	9.81837
hsa03460	Fanconi anemia pathway, *Homo sapiens *(human)	9.53284*E* − 09	53	7.00664*E* − 07	8.020778
hsa05322	Systemic lupus erythematosus, *Homo sapiens *(human)	1.60137*E* − 08	136	9.41604*E* − 07	7.795509
hsa05034	Alcoholism, *Homo sapiens *(human)	5.43613*E* − 07	180	2.66371*E* − 05	6.26471
hsa05204	Chemical carcinogenesis, *Homo sapiens *(human)	1.11406*E* − 06	82	4.67904*E* − 05	5.953093
hsa00983	Drug metabolism, other enzymes, *Homo sapiens *(human)	8.07034*E* − 06	46	0.000296585	5.093108
hsa00980	Metabolism of xenobiotics by cytochrome P450, *Homo sapiens *(human)	0.000014192	74	0.000463605	4.847956
hsa00140	Steroid hormone biosynthesis, *Homo sapiens *(human)	3.3875*E* − 05	58	0.000995926	4.47012
hsa00982	Drug metabolism, cytochrome P450, *Homo sapiens *(human)	6.55397*E* − 05	68	0.001751697	4.183496
hsa04114	Oocyte meiosis, *Homo sapiens *(human)	0.000202216	113	0.004954299	3.694184
hsa03430	Mismatch repair, *Homo sapiens *(human)	0.000264734	23	0.005987061	3.57719
hsa00830	Retinol metabolism, *Homo sapiens *(human)	0.00049597	65	0.01041537	3.304545
hsa04978	Mineral absorption, *Homo sapiens *(human)	0.000531877	51	0.01042478	3.274189
hsa03410	Base excision repair, *Homo sapiens *(human)	0.000844241	33	0.01551292	3.073534
hsa00053	Ascorbate and aldarate metabolism, *Homo sapiens* (human)	0.00090023	27	0.01556869	3.045646
hsa03440	Homologous recombination, *Homo sapiens* (human)	0.001173489	28	0.01916699	2.930521
hsa00500	Starch and sucrose metabolism, *Homo sapiens* (human)	0.001294299	56	0.02002757	2.887965
hsa00240	Pyrimidine metabolism, *Homo sapiens* (human)	0.001511088	105	0.022213	2.82071
hsa03420	Nucleotide excision repair, *Homo sapiens* (human)	0.003406297	47	0.04768816	2.467717
hsa00860	Porphyrin and chlorophyll metabolism, *Homo sapiens* (human)	0.005112004	42	0.06831497	2.291409
hsa05200	Pathways in cancer, *Homo sapiens* (human)	0.006405562	398	0.07929988	2.193443
hsa00040	Pentose and glucuronate interconversions, *Homo sapiens* (human)	0.00647346	36	0.07929988	2.188864
hsa05203	Viral carcinogenesis, *Homo sapiens* (human)	0.006997658	206	0.08229246	2.155047
hsa05222	Small cell lung cancer, *Homo sapiens* (human)	0.00779351	86	0.08812662	2.108267
hsa05219	Bladder cancer, *Homo sapiens* (human)	0.009084523	38	0.09892036	2.041698
hsa05134	Legionellosis, *Homo sapiens* (human)	0.01081157	55	0.1135215	1.966111
hsa04621	NOD-like receptor signaling pathway, *Homo sapiens* (human)	0.0138298	57	0.1402056	1.859184
hsa05202	Transcriptional misregulation in cancer, *Homo sapiens* (human)	0.01531221	179	0.1500597	1.814962
hsa05143	African trypanosomiasis, *Homo sapiens *(human)	0.01618672	34	0.1535128	1.790841
hsa05162	Measles, *Homo sapiens *(human)	0.01985443	134	0.1824125	1.702143
hsa04914	Progesterone-mediated oocyte maturation, *Homo sapiens* (human)	0.02189761	88	0.1950878	1.659603
hsa00410	beta-Alanine metabolism, *Homosapiens* (human)	0.03354188	31	0.2900386	1.474413
hsa00480	Glutathione metabolism, *Homo sapiens *(human)	0.04770932	51	0.4007582	1.321397
