# Supplementary material for: Aberrant Long Noncoding RNAs Expression Profiles Affect Cisplatin Resistance in Lung Adenocarcinoma
Source: Biomed Res Int. 2017 Nov 27;2017:7498151. doi: 10.1155/2017/7498151 (PMC5723956; doi:10.1155/2017/7498151)
Supplement: Supplementary file 1 — Supplemental TABLE 1: Some up-regulated or down-regulated lncRNA in A549/DDP. Supplemental TABLE 2: Some up-regulated or down-regulated lincRNA in A549/DDP and regulation mRNA. Supplemental TABLE 3: Some up-regulated or down-regulated enhancer lncRNA in A549/DDP and regulation mRNA. Supplemental TABLE 4: Some up-regulated or down-regulated antisense lncRNA in A549/DDP and regulation mRNA. [file 7498151.f1.docx]

Supplemental Table 1 Some up-regulated or downregulated lncRNA in A549/DDP

| ProbeName | Fold Change | Log Fold Change | Regulation | GeneSymbol |
| --- | --- | --- | --- | --- |
| ASHGA5P003826 | 4.66411 | 2.2216 | up | HSD17B7P2 |
| ASHGA5P005496 | 4.549855 | 2.1858 | up | RP11-443P15.2 |
| ASHGA5P005611 | 3.832726 | 1.9384 | up | NABP1 |
| ASHGA5P006918 | 2.080162 | 1.0567 | down | RAD1 |
| ASHGA5P008574 | 16.11482 | 4.0103 | up | RP11-327I22.2 |
| ASHGA5P008592 | 3.532166 | 1.8206 | up | ANKRD20A11P |
| ASHGA5P009842 | 2.075759 | 1.0536 | up | TAC3 |
| ASHGA5P010527 | 2.258406 | 1.1753 | up | BLOC1S2 |
| ASHGA5P010584 | 3.93651 | 1.9769 | up | PRKY |
| ASHGA5P011111 | 15.39474 | 3.9444 | up | AL583842.6 |
| ASHGA5P013224 | 5.936566 | 2.5696 | down | RP11-551L14.1 |
| ASHGA5P014028 | 2.900729 | 1.5364 | down | LOC100132774 |
| ASHGA5P014040 | 9.090298 | 3.1843 | down | RP11-551L14.1 |
| ASHGA5P014130 | 2.991225 | 1.5807 | down | ULK4P1 |
| ASHGA5P014184 | 4.009029 | 2.0033 | down | DTYMK |

Supplemental Table 2 Some up-regulated or downregulated lincRNA in A549/DDP and regulation mRNA

| GeneSymbol | Fold change - LncRNAs | LogFold Change | Regulation - LncRNAs | GenomeRelationship | NearbyGeneSymbol | Fold change - mRNAs | Regulation - mRNAs |
| --- | --- | --- | --- | --- | --- | --- | --- |
| XLOC_000086 | 15.9906613 | 3.9992 | up | downstream | RAP1GAP | 3.5856011 | down |
| XLOC_000160 | 2.4131214 | 1.2709 | down | upstream | SCMH1 | 2.8104224 | down |
| XLOC_000160 | 2.4131214 | 1.2709 | down | upstream | SCMH1 | 4.113842 | down |
| XLOC_000160 | 2.4131214 | 1.2709 | down | upstream | SCMH1 | 2.3441494 | down |
| XLOC_000160 | 2.4131214 | 1.2709 | down | upstream | SCMH1 | 2.2471227 | down |
| XLOC_000429 | 3.3290289 | 1.7351 | up | downstream | CD1B | 2.165553 | up |
| XLOC_000447 | 4.8134089 | 2.2671 | down | upstream | NUF2 | 9.5849473 | down |
| XLOC_000704 | 2.3430277 | 1.2284 | up | downstream | MAD2L2 | 3.2135301 | down |
| XLOC_000737 | 19.8164111 | 4.3086 | up | upstream | FUCA1 | 2.2264728 | up |
| XLOC_000833 | 2.1152615 | 1.0808 | down | downstream | CDKN2C | 6.3987682 | down |
| XLOC_000833 | 2.1152615 | 1.0808 | down | upstream | C1orf185 | 3.2869732 | up |
| XLOC_000932 | 2.4889634 | 1.3155 | down | downstream | DPYD | 5.4609105 | down |
| XLOC_000951 | 5.0415757 | 2.3339 | up | downstream | WDR47 | 2.2574928 | up |
| XLOC_000951 | 5.0415757 | 2.3339 | up | downstream | TAF13 | 2.3021707 | up |
| XLOC_000951 | 5.0415757 | 2.3339 | up | downstream | GPSM2 | 4.6489503 | down |

Supplemental Table 3 Some up-regulated or downregulated enhancer lncRNA in A549/DDP and regulation mRNA

| GeneSymbol | Fold change LncRNAs | LogFold Change | Regulation LncRNAs | GenomeRelationship | NearbyGeneSymbol | Fold change mRNAs | Regulation mRNAs |
| --- | --- | --- | --- | --- | --- | --- | --- |
| LINC00475 | 2.1070908 | 1.0753 | up | downstream | CENPP | 2.7764405 | down |
| RP11-314N13.3 | 2.5808745 | 1.3679 | up | upstream | SDCBP2 | 2.4930806 | down |
| RP11-396C23.2 | 6.4252407 | 2.6838 | up | downstream | LIN9 | 3.3128452 | down |
| RP11-402G3.5 | 3.8893956 | 1.9595 | down | upstream | TNFSF15 | 12.0302899 | up |
| JPX | 3.6737753 | 1.8773 | up | upstream | CHIC1 | 2.6234591 | up |
| RP11-143M1.4 | 15.9152289 | 3.9923 | down | upstream | C9orf66 | 22.5586292 | up |
| RP11-143M1.4 | 15.9152289 | 3.9923 | down | upstream | DOCK8 | 2.6391567 | down |
| RP11-143M1.4 | 15.9152289 | 3.9923 | down | upstream | CBWD1 | 3.3228381 | up |
| RP11-143M1.4 | 15.9152289 | 3.9923 | down | upstream | CBWD1 | 3.8093768 | up |
| RP11-143M1.4 | 15.9152289 | 3.9923 | down | upstream | FOXD4 | 4.2006599 | up |
| RP11-554I8.2 | 3.2692733 | 1.7090 | up | upstream | PRKCQ | 2.0416557 | up |
| RP11-533K9.2 | 2.0080915 | 1.0058 | up | downstream | C9orf3 | 2.4782158 | down |
| RP11-533K9.2 | 2.0080915 | 1.0058 | up | downstream | C9orf3 | 2.4215195 | down |
| RP11-54O7.3 | 2.4122207 | 1.2704 | up | upstream | HES4 | 2.5471897 | down |
| RP11-54O7.3 | 2.4122207 | 1.2704 | up | upstream | HES4 | 2.3562751 | down |
| RP11-54O7.3 | 2.4122207 | 1.2704 | up | upstream | TNFRSF18 | 2.9657994 | down |
| RP11-54O7.3 | 2.4122207 | 1.2704 | up | upstream | AGRN | 2.0439005 | up |

Supplemental Table 4 Some up-regulated or downregulated antisense lncRNA in A549/DDP and regulation mRNA

| GeneSymbol | Foldchange LncRNAs | LogFold Change | Regulation LncRNAs | Genome Relationship | | NearbyGene Symbol | Fold  change mRNAs | Regulation  mRNAs |
| --- | --- | --- | --- | --- | --- | --- | --- | --- |
| RP11-315I20.1 | 4.5024673 | 2.1707 | up | | intronic antisense | LIX1L | 2.237578 | down |
| RP11-277A4.4 | 2.2632689 | 1.1784 | down | | intronic antisense | TMEM39B | 3.3033412 | down |
| AC114812.5 | 2.192798 | 1.1328 | up | | intronic antisense | UGT1A6 | 6.5094254 | up |
| AC114812.5 | 2.192798 | 1.1328 | up | | intronic antisense | UGT1A8 | 4.3597911 | up |
| AC114812.5 | 2.192798 | 1.1328 | up | | intronic antisense | UGT1A3 | 3.4602397 | up |
| AC114812.5 | 2.192798 | 1.1328 | up | | intronic antisense | UGT1A9 | 2.1380965 | down |
| AC114812.5 | 2.192798 | 1.1328 | up | | intronic antisense | UGT1A6 | 3.5721283 | up |
| RP1-283K11.3 | 2.1089794 | 1.0765 | up | | intronic antisense | EYA4 | 14.0135182 | down |
| RP1-283K11.3 | 2.1089794 | 1.0765 | up | | intronic antisense | EYA4 | 23.4333563 | down |
| RP1-283K11.3 | 2.1089794 | 1.0765 | up | | intronic antisense | EYA4 | 6.2333555 | down |
| RBMS3-AS3 | 7.7360783 | 2.9516 | down | | intronic antisense | RBMS3 | 14.4131024 | down |
| RBMS3-AS3 | 7.7360783 | 2.9516 | down | | intronic antisense | RBMS3 | 2.2781236 | up |
| RBMS3-AS3 | 7.7360783 | 2.9516 | down | | intronic antisense | RBMS3 | 8.5131763 | down |
| RP4-694A7.4 | 5.9611117 | 2.5756 | down | | intronic antisense | DEPDC1 | 3.2655554 | down |
| RP4-694A7.4 | 5.9611117 | 2.5756 | down | | intronic antisense | DEPDC1 | 8.3703422 | down |
| DLG3-AS1 | 2.3413756 | 1.2274 | down | | intronic antisense | DLG3 | 2.2609505 | down |
